# Authors’ Response to Peer Review of “Use of Mobile Forms in Low-Resource Areas for Population Health Surveys: Interview and Field Test Study”

**DOI:** 10.2196/79539

**Published:** 2025-08-11

**Authors:** Alexander Davis, Aidan Chen, Milton Chen, James Davis

**Affiliations:** 1 University of California, Santa Cruz, CA, United States; 2 VSee Health, Newton, MA, United States; 3 Department of Computer Science and Engineering, University of California, 1156 High St, MS:SOE3, Santa Cruz, CA, 95064, United States, 1 (831) 459 1841

**Keywords:** mobile forms, offline forms, electronic data capture, design, low-resource settings, health surveys

*This is the authors’ response to the peer-review report for “Use of Mobile Forms in Low-Resource Areas for Population Health Surveys: Interview and Field Test Study.”*

## Major Concerns and Feedback

*Rationale of the approach: Reviewers [1] had some questions about the rationale behind the choice of the approach. Was there an initial hypothesis that was tested? If so, can the authors explain the rationale in more detail?*

**Response:** The paper [2] was modified so that this was discussed in more detail in the Introduction section.

*General clarity: The language used was straightforward, with simple and short sentences, so was generally very easy to follow. However, several reviewers found the manuscript very descriptive and lacking critical analysis/reflection (more on this later in the review). Furthermore, some parts of the article could benefit from restructuring the text (moving text to different sections). For example, it is recommended that the authors consider moving the findings described in the Methodology section to the Results section. Authors may also consider streamlining the manuscript to ensure the same result is not repeated multiple times in the same section, which can be confusing for the reader.*

**Response:** The paper was modified so that major sections of the paper were restructured to adhere to JMIR Publications guidelines. Methodology findings were also moved to the Results section. We also streamlined the manuscript to ensure that we didn’t repeat anything that was mentioned before.

*More methodological details: While the study outlines the general approach used in the pilot interviews and field testing, it would be helpful to add detailed methodological specifics, like the criteria for selecting survey sites and surveyors, the precise training process for surveyors, the number and conditions of interviews, the demographic of the population tested, and the kind of interview method that was used.*

**Response:** The paper was modified so that, where needed, we added extra information (eg, the training process for surveyors and the criteria for selecting survey slots).

*Descriptive results, vague language, unsupported conclusions: The interpretation of the data seems primarily positive toward mobile forms, but it might be somewhat biased due to the lack of objective measures and control groups—the conclusions are largely based on subjective feedback rather than on a comprehensive analysis of performance metrics. This is an important limitation of the study that should be at least recognized. For example, the sentence “The surveyors mostly used their phones for Social Media and Messaging apps. This indicated that these surveyors were reasonably comfortable using their phones.” is a conclusion based on general observation rather than on quantitative assessment. Another example: “Surveyors interviewed were chosen through convenience sampling”; what did the authors mean by this? More information would be needed to better understand how the selection of surveyors was done.*

**Response:** The paper was modified so that for some of these points we have added clarification, and for others included some discussion in the Limitations section.

*More technical information: The study doesn’t provide in-depth information about the technical aspects of the mobile form software (eg, what language was used to write the code, the code itself). Without this information, replicating the software for a similar study would be challenging. If readers are unable to access the source code used to generate the software, the reproduction and validation of the results would not be possible. The reviewers suggest that the authors consider sharing the source code on GitHub with an open-source license so that others are able to investigate the code, build upon it, and adapt it to their needs so that other groups with the same issues can benefit from this work too.*

**Response:** The paper was modified so that more technical information about the software, which was previously omitted, is now placed under the Methodology’s Survey Form Software section.

*Ethics and privacy: Reviewers had several concerns about ethical and privacy issues related to the study. They asked if the mobile app was Health Insurance Portability and Accountability Act compliant and if it had obtained institutional review board approval. Furthermore, the reviewers expressed concern about data privacy for the people who were surveyed through the app. Where were the data stored? Were there ways to secure the data collected on private phones so that they could not be stolen easily?*

**Response:** The paper was modified so that the ethics and privacy was addressed in the Ethical Considerations section of the paper. Technical details were also mentioned in the Survey Form Software section under Methodology.

*Study limitations: Reviewers identified several limitations of the study and suggest that they be discussed in a separate section of the Discussion so that the reader can easily access them. The most important limitations include geographic and demographic limitations, sample selection, lack of a control group, potential technological familiarity and bias (eg, are the people developing the tool the same as the ones conducting the survey?), depth of usability testing, and software development process. Furthermore, although the findings show that there is a dominant interest in mobile forms, the issue of lack of phone ownership, poor internet access, typing speed, and the educational status of the participants should be properly discussed.*

**Response:** The paper was modified so that a section to discuss the limitations of our research was added. All of the reviewers’ concerns are addressed in that section.

## Minor Concerns and Feedback

*Software like REDCap and SurveyMonkey can work offline and can time questions. It would be helpful to compare this newly developed software with existing ones with comparable features.*

**Response:** The paper was modified, so under the Methodology’s Pilot Interview section, we mentioned why REDCap and SurveyMonkey wouldn’t work for our situation as it didn’t have every feature we needed.

*Some reviewers wondered if the authors quantified differences in the degree of numerical literacy, language literacy, and technological literacy among the surveyors as factors that could have influenced the speed of filling the mobile forms.*

**Response:** The paper was modified so that some clarification was added, but this question is about something we did not consider in our study and thus cannot report on.

*One of the findings was that a portion of the surveyors were not found to be proficient with modern technology. Some reviewers wondered if the authors saw a correlation between technological proficiency and age. It would be interesting to show if that was the case.*

**Response:** The paper was modified, so under the Methodology’s Pilot Interviews section, we mentioned that we found a minor correlation.

*It would be helpful to know whether informed consent was obtained from the surveyors.*

**Response:** The paper was modified so that a section in the paper talking about ethics/consent for the research was added.

*More information about the research conditions in this context would be helpful (high school internship in the company). Also, sentences like the following one don’t help the reader understand the scientific context or topic: “Since Gawad Kalinga builds free housing in ten thousand locations across the Philippines, it can reach over one million households and mobilize many volunteers.” The reviewers suggest that authors more clearly and specifically state what they want to communicate, in that case presumably that the partner wants to reach respondents on a bigger scale.*

**Response:** The paper was modified so that we reworded and added some more text to describe our end goals more clearly.

*The reviewers praise the data visualization, as the authors made it easy for readers to grasp the results. However, higher image resolutions would help improve Figures 1 and 3. Some wondered how Figure 1 supports the argument.*

**Response:** Figure 1 is there to show the deployment environment. These images will be uploaded at the appropriate resolution.

*It would be helpful to have a table summarizing the characteristics of participants.*

**Response:** The paper won’t be modified because most of the characteristics data that was collected was removed during anonymization; thus, we lack enough data to create a meaningful table.

*In the Introduction, the authors mention there were 33 surveyors, but in the figures, it looks like there were 50.*

**Response:** The paper was modified so now we have added clarification that there were 53 surveyors in our pilot study, but 20 didn't continue until the end of this study, so they were excluded from the final analysis, leading to a different participant count at different stages.

*Figure 2 should be under the Results section instead of Methods.*

**Response:** The paper was modified so now we separated Pilot Interview into two sections: Methodology and Results. We also moved Figure 2 to the Results’ Pilot Interview Analysis section.

*Figure 2 and several subsequent ones: The captions should describe the figures and not interpret the results. Interpretation of the results should be reserved for the Results section (to a certain extent) and for the Discussion.*

**Response:** The paper was modified so that the caption was changed to just describe the figures, and the interpretation is now moved to the Results section.

*If data are comparable, it would be useful to have pre- and postpreference for mobile forms presented in the same figure for comparison, perhaps using different colors for clarity.*

**Response:** The paper was modified so that the pre-preference for mobile forms was added below the post-preference.

*In Figure 4, regarding the location of the study, it would be better either in the Introduction section or primary paragraphs of the Methodology.*

**Response:** The paper was modified so that Figure 4 is now moved to the Introduction section and renamed Figure 1.

*In Figure 6, histogram and summary statistics in text could supplement the visualization.*

**Response:** The paper was modified, so generally, the text was edited to make this more clear.

*It would be important to include how many surveyors were interviewed right at the beginning of the Methodology section rather than waiting until later in the manuscript.*

**Response:** The paper was modified, so in the Methodology’s Pilot Interviews section, we mentioned the surveyor count in the first paragraph.

*Were there any problems regarding the battery life/charging of the mobile phones? How was this dealt with? Were surveyors provided with a charged power bank to overcome a potential lack of power?*

**Response:** The paper was modified, so under the Methodology’s Field Testing section, we added a paragraph about battery life and how it wasn’t a big issue.

*A reviewer suggested the addition of a voice command to the digital survey as a way to collect qualitative research not only for research questions in future research, like open-ended and closed-ended questions.*

**Response:** The paper was modified, so under the Methodology’s Field Testing section, we added a paragraph about battery life and how it wasn’t a big issue.
